# Constitutive relationship between pore-permeability and molecular structure of bituminous coal based on the Kozeny-Carman equation

**DOI:** 10.1371/journal.pone.0327790

**Published:** 2025-07-15

**Authors:** Mingkun Pang, Tianyu Zhang, Lin Li, Tianjun Zhang, Hongyu Pan

**Affiliations:** 1 College of Safety Science and Engineering, Xi’an University of Science and Technology, Xi’an, China; 2 Key Laboratory of Western Mine Exploitation and Hazard Prevention of the Ministry of Education, Xi’an, China; Wadia Institute of Himalayan Geology, INDIA

## Abstract

The cell structure of three different densities of bituminous coal molecules was modeled and optimized by using Material Studio (MS) to reveal the mathematical relationship between the porosity and permeability of bituminous coal. The internal surface area was analyzed using the Connolly Surface module, and the specific surface area of the molecules was obtained by mass conversion; the grey processing method was used to evaluate the cell structure porosity, and the pore-permeability constitutive relationship of bituminous coal molecules was established by drawing on the Kozeny-Carman (KC) equation. The results show that the maximum value of 0.29 appears near the pore size of 2.01 Å; Porosity is the main factor affecting its permeability, and the permeability decreases with increasing porosity. The relationship between porosity and permeability was obtained by regression analysis as follows: *K* = 161 ϕ ^2.1^/(1 + 12 ϕ ^2.1^). This study provides a new method for the calculation of permeability of porous media.

## 1 Introduction

Coal is an important source of energy and is fundamental to the needs of any economy and the society it supports. Permeability and porosity are two important basic parameters for efficient extraction of coal bed gas, and both are closely related to the pore structure of coal [1,2]. The pore structure of coal, includes pore size distribution (PSD), pore volume, specific surface area, pore shape, *etc.* At present, methods to study the pore structure of coal include high-pressure mercury injection, scanning electron microscopy, and nuclear magnetic resonance [3,4], and significant research results have been achieved in terms of PSD, shape, and fractal characteristics. The pore nature of coal is the main factor affecting the physico-chemical properties of coal, which directly affects the efficient extraction of gas from coal seams, carbon dioxide storage, *etc*., and the study of the pore structure of coal is necessary for the efficient collection of gas resources [5].

Current research of pore structure properties of coal is mainly focused on two topics: accurate determination of permeability of coal and molecular simulation. For the accurate determination of permeability and porosity of such media, in 1937, Kozeny (1927) and Carman (1938) improved the calculation of Darcy’s permeability *K* and proposed the KC equation [6,7]. At present, the KC equation has been widely accepted and used in many engineering scenarios, but it has many limitations revealed since its first use. The reason for this is that the equation is semi-empirical relationship, and the KC constant is empirical, proven not to be constant, and perhaps related to the porosity [8]. To gain insight into the microscopic pore structure of coal, Li *et al.* [9] investigated the pore parameters using small-angle X-ray scattering and found that the microporosity was indeed correlated with the fractal dimension. Xiao *et al.* [10] concluded that fractal theory can be invoked to analyze the complex pore structure and that the sandstone pore system is mainly composed of macroporosity and microporosity. From the fractal dimension and NMR curve characteristics, the low-order coal seepage pores are dominated by macropores. Wyrzykowski *et al.* [11] studied the pore structure using MIP and concluded that the porosity was significantly increased by applying mortar to a substrate with high water absorption. MIP has its limitations, as demonstrated by Bhuiyan *et al.* [12] in measuring the true PSD, which has been confirmed by researchers working with porous materials. Considering the microstructure, coal is a complex material consisting of inorganic and organic materials with complex internal surface structural features. Determining the mechanism of coal-gas interaction from the molecular level has been a fundamental problem in CBM research and development [13,14]. The mechanism of microscopic pore evolution between coal molecules was studied at the molecular level using computerized molecular simulations [15]. Hu *et al*. [16] and You *et al.* [17] investigated the self-diffusion and inter-diffusion processes of CO_2_ and CH_4_ multi-component gases by molecular simulation. Sun *et al.* [18] developed a new MS-BPD molecular pore model to study the interactions of multi-component gases in coal molecules during gas injection. Sun *et al.* [18] established a new MS-BPD molecular pore model to study the diffusion of multi-component gases in the molecular pore structure of coal during gas injection, and evaluated the effect of multi-component gas injection on the gas recovery rate. Therefore, molecular simulation as a theoretical research method has also received wide attention in the study of the pore structure of coal [19–21].

To analyze the pore structure characteristics of coal from the molecular level perspective, in the present study, the pore characteristic parameters of bituminous coal were determined using molecular simulation based on the relationship between porosity and permeability given in the KC equation. By establishing molecular cells with different densities, the lowest energy structure model of coal was developed by annealing in dynamics simulations of the coal-structure model using Anneal in the Forcite module, and then the permeability variations in different molecular structures were assessed. The relationship between pore structure and permeability was then revealed. This work provides a theoretical basis for the efficient extraction and collection of coal seam gas.

## 2 Theoretical background

Coal is a porous medium rich in pore and fracture structures. The permeability of this porous medium is mainly determined by the size and dimensions of its pores and fractures, so most scholars studying the porosity of this medium mainly focus on finding the best method of calculation of the permeability.

### 2.1 Definition of pore-permeability

**Fig 1:**
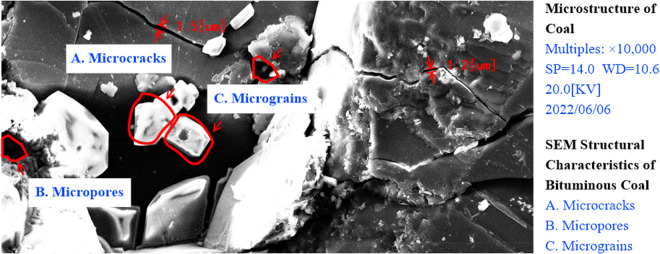
Microscopic porous structure of bituminous coal under SEM.

From the physical structure point of view, coal is a solid material with complex pore structure, and the physical structure varies greatly between different coal types and formations, *e.g*., the density of bituminous coal can be between 1.1 and 2.3 g/cm^3^, and the porosity varies between 0.2 and 0.9. The pore structure of bituminous coals (above the nanoscale) could be observed by using scanning electron microscopy (SEM) [22,23]. The microstructure of coal under SEM scanning is given in Fig 1.

The gap between the particles that make up the crushed body is called the pore space. Taking the total volume of the crushed body as l, the pore volume expressed as a percentage of the total volume is called the porosity. The porosity of the crushed body is related to its particle gradation (*i.e*., the relative content of various sizes of lumpy particles), the shape and mutual position of the particles and the pressure to which they are subjected. The ratio of the pore volume to the volume of the solid block is called the pore ratio. There is a relationship between porosity and pore ratio as follows:


$$\phi =\frac{\varepsilon }{1+\varepsilon }\times 100\;\%\;,\;\varepsilon =\frac{\phi }{1+\phi }\times 100\;\%$$ (1)

where ϕ denotes the porosity and *ε* is the porosity ratio.

### 2.2 Calculation of pore-permeability

Permeability is a fundamental parameter describing the ability of a fluid to pass through a porous medium, which is influenced only by the structural characteristics of the medium itself and is independent of the properties of the fluid itself [24,25]. The Kozeny-Carman (KC) equation is the best-known permeability-porosity relationship, which is widely applied in the field of percolation in porous media and is the starting point for many other permeability models. The KC equation relates the permeability *k* directly to porosity ϕ [26,27].


$$K=\frac{\phi^{3}}{C{\left(1-\phi \right)}^{2}S^{2}}$$ (2)

where *C* denotes the Kozeny constant and *s* denotes the specific surface area, however, *C* is an empirical parameter related to the initial porosity of the medium. For fractured coal media [28], Carman has shown that its value is approximately equal to 5.


$$K=\frac{\phi^{3}}{C{\left(1-\phi \right)}^{2}S^{2}}=\frac{\phi^{3}}{36C{\left(1-\phi \right)}^{2}}d^{2}$$ (3)

where: *d* is the average equivalent spherical diameter of the particles (after Pang *et al.* [29]) whose value can be taken as *d*_50_ from the grading curve.

## 3 Simulation experiments

To study the permeability characteristics of bituminous coal molecular structure, the lowest energy structure model of coal was established by setting different initial densities and by using the Anneal feature in the Forcite module to simulate the annealing kinetics of the coal structure model, and then the variations in permeability across different molecular structures were determined.

### 3.1 Molecular model

**Fig 2:**
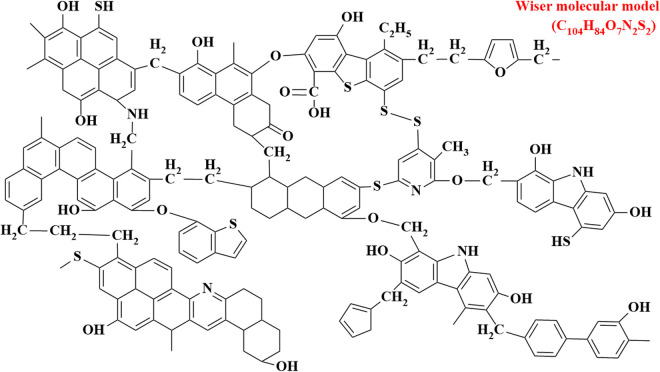
The Wiser molecular model of bituminous coal.

The study of molecular models of coal first started in the 1950s, and in 1942, Fuchs built the first lignite model, which laid a solid foundation for the development of structural models of coal. In 1975, the Wiser model added the ether bond between aromatic ring structures to the original one, which can basically reflect the mechanism of sulfur-containing compounds production in coal during combustion and the molecular structure characteristics of bituminous coal [30,31]. Since the most suitable coal for CO_2_ sequestration is bituminous coal or sub-bituminous coal, a molecular model of bituminous coal (C_104_H_84_O_7_N_2_S_2_) was developed based on a published model [32]. The Wiser molecular model of bituminous coal is shown in Fig 2.

From Fig 2, it can be seen that the molecular model of bituminous coal contains C, H, O, N, and S, including 81.25% carbon, 5.34% hydrogen, 4.17% sulfur, 1.82% nitrogen, and 7.28% oxygen. It is consistent with the bituminous coal composition measured under experimental conditions [33].

The industrial and elemental analysis of coal is a fundamental part of coal quality analysis and is closely related to the genesis, kerogenicity, and petrographic composition of coal. The results of industrial and elemental analyses of the three samples are summarized in Table 1.

**Table 1:** Industrial analysis and elemental analysis results of bituminous coal.

Coal sample number	Industrial Analysis, ω(%)			Industrial Analysis, *w*(%)(daf)				
	Mad	Aad	Vad	C	H	O	N	S
DFS-4#coal	1.02	10.45	6.85	87.19	3.42	8.523	0.71	0.157
WJP-4#coal	9.88	8.47	33.28	72.49	3.80	21.53	0.76	1.423
JJH-4#coal	7.55	8.81	33.24	73.82	4.42	19.40	0.72	1.631

In order to explore the impact of density on the molecular structure of bituminous coal, we have established molecular models with three different initial densities, and the bituminous coal model molecules were numbered Alpha-1, Alpha-2, and Alpha-3. The initial densities of these three oxidized bituminous coals were set to 1.00 g/cm^3^, 1.20 g/cm^3^, and 1.40 g/cm^3^. Annealing in our molecular dynamics simulations was also used to optimize the structures.

### 3.2 Simulation methodology

Material Studio is a molecular simulation software developed by the world’s leading BIOVIA computational science company, which integrates Quantum Mechanics, Molecular Mechanics, Molecular Dynamics, and Monte Carlo simulation. It can calculate and visualize the kinetic trajectories of changes in molecular energy.

**Fig 3:**
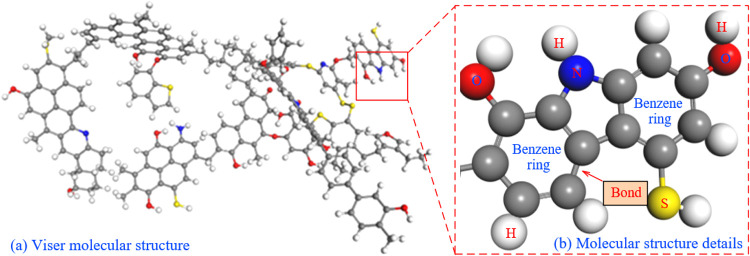
Changes in porosity of the molecular structure after rupture.

In this study, the free pore volume of bituminous coal before and after oxidation was calculated using the atomic volume insertion probe method. The free pore volume was detected using probe molecules with fixed connection radii [34], which were randomly inserted into the established model system and rolled over the van der Waals surfaces of bituminous coal and bituminous coal structures after oxidation. Thus, the surface of the solid structure was determined and the area wrapped around the surface of the structure was identified as the free pore volume. Changes in the porosity of the molecular structure after rupture are shown in Fig 3.

The porosity of this molecular model can be obtained by calculating the ratio of the free volume of the model to the crystal volume, and the Connolly Surface tool in Material Studio software can construct a molecular model Connolly surface using gas molecules as probes around the molecule and find the free volume of the model.

The comparisons of microcrystalline structure, density and porosity between the actual coal sample and the molecular model show that the physical properties of this molecular model are similar to those of the actual coal sample, so the constructed BN-2 complex molecular model is reasonable and can be used for a series of subsequent simulation experiments.

### 3.3 Molecular mechanics

According to the Bonn-Oppenheimer approximation, the potential energy of a molecule can be considered as a function of the spatial coordinates of the individual atoms comprising the molecule. This potential energy function, which describes the relationship between molecular energy and conformation in mathematical form, is called the molecular force field. The molecular force field function system consists of two parts: one is the potential energy function used to link the molecular configuration and energy; the other is a set of physical parameters (bond length, bond angle, and dihedral angle) of each atom in different bonding situations, which are generally measured experimentally or calculated by quantum chemistry. The potential energy functions generally include the following three items.


$$E_{total}=+E_{valence}+E_{non-bond}+E_{crossterm}$$ (4)

where: *E*_valence_ denotes the bond energy, *E*_no-bond_ is the non-bond energy between atoms, and *E*_cross-term_ is the covalent cross-bond energy.

The Amorphous Cell module is a tool for constructing amorphous models based on the molecular mechanics method and using the Monte Carlo method for calculations. This module is an important aid to the simulation work in the field of porous materials and is applied in this study to add periodic boundary conditions to the constructed molecular model of coal.

The COMPASS force field is chosen in a functional form to ensure the accuracy of the energy calculation while keeping the calculation simple. The choice of force field is crucial in molecular simulations and is generally based on the following three objectives: (1) to reproduce the structural properties of the molecule; (2) to predict certain specific properties, which is the basis for the parameterization of the force field; and (3) to describe the inter- and intra-molecular forces of all molecules of a certain type more accurately. The force field chosen in the present work is described below. The Forcite module, with its diverse algorithms and ability to optimize structural models, is a more desirable module for use in molecular mechanics calculations. The module can perform potential energy and geometry optimization calculations for arbitrary molecular and periodic systems, as well as fast energy calculations, geometry optimization and dynamics simulation studies under various system syntheses for molecular, surface or 3-d periodic material systems.

In the present work, this module was used to perform geometry optimization of gas molecular models, as well as annealing simulations to obtain the lowest energy conformation of molecules; and molecular dynamics simulations of the adsorbed system were also made with a view to obtaining the interaction between gas molecules and coal surfaces.

## 4 Results and discussion

In this study, the effect of pore structure on the permeability of coal body was analyzed by modelling bituminous coal molecules with different densities. The pore surface distribution characteristics of bituminous coal molecules were investigated to reveal the effect of molecular density on the porosity; the relationship between pore structure and permeability was revealed, and its corresponding functional expressions were presented.

### 4.1 Distribution characteristics of molecular pore surfaces

**Fig 4:**
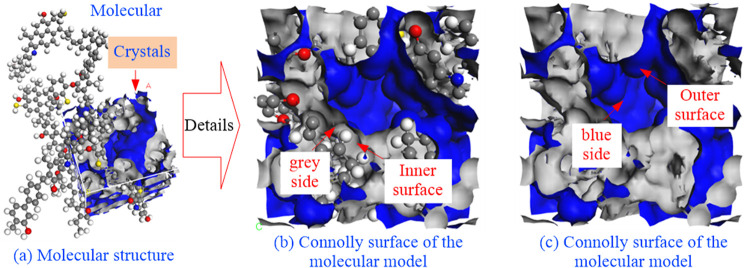
Schematic diagram of the inner and outer surfaces of Connolly for the molecular model.

The molecular probe used in this measurement is a CH_4_ molecule with a kinetic diameter of 0.38 nm. The figure below shows the Connolly surface of the molecular model, which is composed of several small cavities. The free volume of the model is 356.58 Å^3^ by adding up the volume of these cavities, and the total volume of the model is 2771.55 Å^3^. The calculation shows that the porosity of the model is 12.87%, which is slightly larger than that measured; the difference between the two is small, so the pore structure of the model can replace the actual coal sample. The schematic diagram of the Connolly inner and outer surfaces of the molecular model is illustrated in Fig 4.

In this simulation, two molecules were placed into one cell, and the structural characteristics of the molecules within the cell were studied by the statistical method inherent to the Connolly Surface tool in MS.

**Fig 5:**
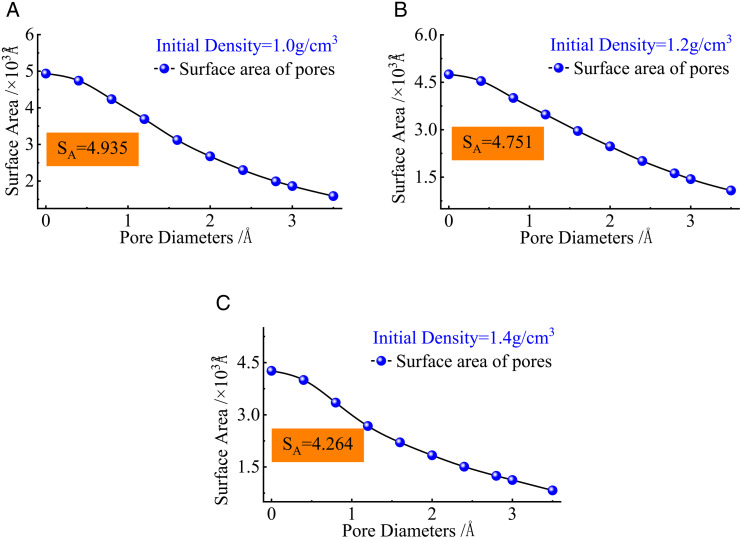
PSD curves of cells at different densities. **(a)** Alpha-1 group of test values, **(b)** Alpha-2 group of test values. **(c)** Alpha-3 group of test values.

In addition, a series of spherical probes with gradually increasing connection radius were used to detect the corresponding free pore volume and a ratio was made with the total pore volume of bituminous coal measured when the probe diameter was zero, and the PSD of bituminous coal was obtained by differentiating this ratio with respect to the corresponding probe diameter. The PSD curves of the cells at different densities are demonstrated in Fig 5.

In Fig 5, the first test set was undertaken at an initial density at 1.0 g/cm^3^, and the maximum surface area in the test result is 4.935 × 10^3^ Å^2^, which is the maximum surface area across the three densities tested. In the second test, the initial density is set to 1.2 g/cm^3^ and the maximum surface area is 4.751 × 10^3^ Å^2^. In the third test, the initial density is set to 1.4 g/cm^3^ and the maximum surface area is 4.426 × 10^3^ Å^2^. The peak of the PSD curve decreases as the density of bituminous coal increases. This indicates that as the density increases, the proportion of bituminous coal in the small pore size increases and the proportion in the large pore size decreases. By analyzing the micropore structure of coal molecules at mesoscale, on the one hand, it can be found that there are the most likely connecting trend points between adjacent pores and pores, and master the evolution law of micropore structure with large – medium – small pores.

**Fig 6:**
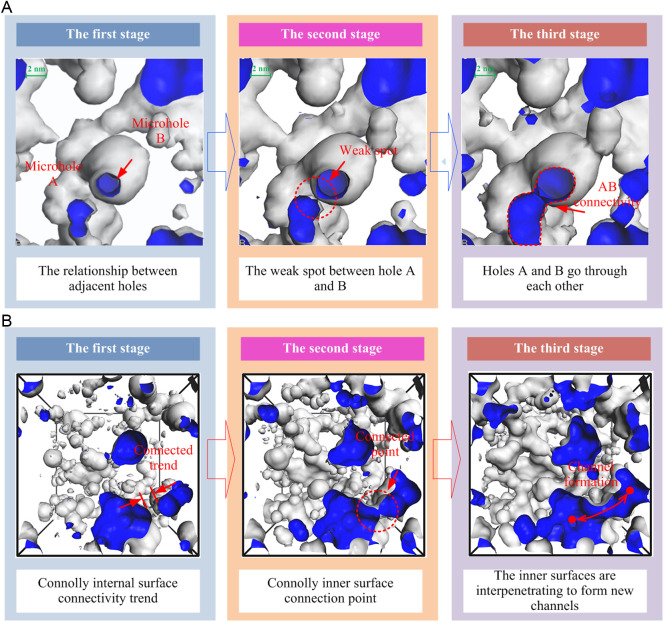
Diagram of evolution process of molecular micropore structure of coal.

In addition, as the diameter of the probe molecule changes in size, the holes that can be measured are different. As the diameter of the probe molecule increases, fewer and fewer pores can be found; the smaller the probe molecule, the more pores that we can detect. Therefore, visible and invisible pores are relative, and certain pores that can be detected by small diameter probes but not by large probes are relatively invisible pores. Probes of different diameters were used to detect pores in the three-dimensional structure of two coal samples. **The Fig 6. visually shows the pore evolution and connectivity characteristics during the detection process.**

### 4.2 Effect of molecular density on porosity

The potential energy surface of coal macromolecule structure is complex, and after optimization by molecular mechanics, only the local minimum value of energy was obtained, not the minimum value of the whole potential energy surface. The lowest energy structure model of coal can be determined by annealing, which reaches the minima in local energy and overall energy. The Anneal function in the Forcite module can be chosen to simulate the annealing kinetics of the coal structure model.

**Fig 7:**
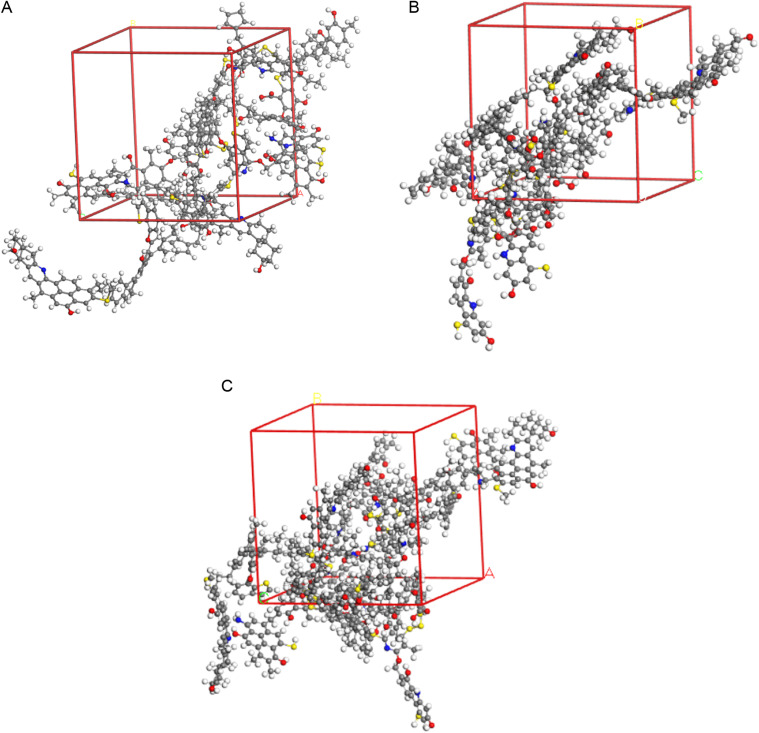
Density distribution of optimized bituminous coal crystals. **(a)** Density = 1.127 g/cm^3^. **(b)** Density = 1.156 g/cm^3^. **(c)** Density = 1.165g/cm^3^.

The optimized density distribution of the bituminous coal cells is shown in Fig 7. To investigate the effect of density variation on the pore structure of bituminous coal, the molecular model of the oxidized bituminous coal established in the present work, three structural models of bituminous coal with three densities were established, annealing and molecular dynamics simulations for the models were also undertaken to optimize the structure, the final densities of these three bituminous coals are 1.127 g/cm^3^, 1.156 g/cm^3^, and 1.165 g/cm^3^, respectively.

**Fig 8:**
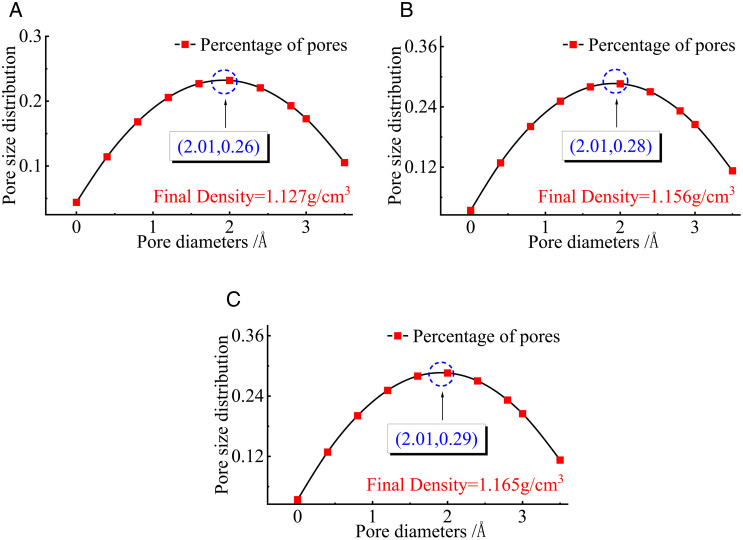
PSD of cells at different densities. **(a)** Alpha-1 group of test values. **(b)** Alpha-2 group of test values. **(c)** Alpha-3 group of test values.

The PSDs of the cells at different densities are displayed in Fig 8. Similarly, the PSD profiles of the amorphous cell structures of these three bituminous coals after oxidation without density were calculated using the atomic volume insertion probe method.

As illustrated Fig 8, the PSD is located at (2.01, 0.26), (2.01, 0.28), and (2.01, 0.29). The peak of the PSD curve increases with increasing density of bituminous coal molecules, and the maximum value of 0.29 appears near the pore size of 2.01 Å. The values of the PSD curve increase with increasing density before the peak and decrease with increasing density beyond the peak. This indicates that the proportion of pores in the oxidized bituminous coal increases at the smaller pore size and decreases at the larger pore size as the density increases.

In order to further assess the pore characteristics of bituminous coal molecules, the calculation of specific surface area is defined as follows:


$$S=\frac{s_{surfacearea}}{m}=\frac{s_{surfacearea}}{V\cdot \rho_{finaldensity}}$$ (5)

where: *S* is the specific surface area based on molecular volume; *m* denotes the actual mass of the molecule; *V* represents the total volume of the molecule; $s_{surfacearea}$ is the Connolly surface area of the molecule. $\rho_{finaldensity}$ is the final density of the molecule after optimization; molecular pore structure parameters were calculated as summarized in Table 2.

**Table 2:** Molecular pore structure: parameter calculation table.

Parameter determination	Pore detail parameters at different densities		
	Alpha −1	Alpha −2	Alpha −3
Cell size L × W × H (Å)	23.5 × 23.5 × 23.5	21.8 × 21.8 × 21.8	19.5 × 19.5 × 19.5
Initial occupied volume (Å^3^)	5.391 × 10^3^	5.399 × 10^3^	5.356 × 10^3^
Initial surface area (Å^2^)	4.935 × 10^3^	4.751 × 10^3^	4.264 × 10^3^

### 4.3 Relationship between pore space and permeability

The disorderly nature of the pore microstructure suggests the presence of fractal features formed by pore and capillary bending. Katz *et al.* [35] may be the first to present experimental evidence that the pore space of a set of samples is fractal, with lengths ranging from 10 Å to 100 μm being self-similar across three to four orders of magnitude.

#### (1) Pore fractal characteristics.

**Fig 9:**
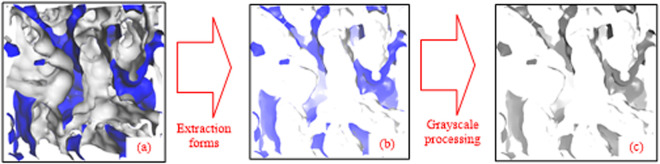
Extraction process of molecular structure pore parameters of bituminous coal within the crystal cell.

Fractal geometry theory has been shown to be a powerful tool for studying porous media with complex and random microstructures [36], and several fractal geometric models have been proposed for the permeability coefficients of porous media [37]. The fractal dimension *D*_f_ is such that 1 < *D*_f_ < 2 and 2 < *D*_f_ < 3 in two and three dimensions, respectively. Yu *et al.* [38] applied the theory of fractal geometry to propose the cumulative size distribution law of pores in porous media. The extraction process of the pore parameters of the bituminous coal molecular structure within the crystal cell is depicted in Fig 9.

The fractal dimension of the pore space can be deduced from Equation (3) for the total number of pores varying from the minimum diameter *k*_min_ to the maximum diameter *k*_max_:


$$N(\varepsilon \geq \lambda )=\;{\left(\frac{\lambda_{\max}}{\lambda }\right)}^{D_{f}},\;N\left(\varepsilon \geq \lambda_{\min}\right)=\;{\left(\frac{\lambda_{\max}}{\lambda_{\min}}\right)}^{D_{f}}$$ (6)

where: *N* is the number of pores, *ε* denotes the length scale, and *λ* represents the pore size. Differentiation of *k* yields the number of pores with sizes in the infinitesimal range *k* to *k* + δ*k*.


$$-dN=D_{f}\lambda_{\max}^{D_{f}}\lambda^{-\left(D_{f}+1\right)}d\lambda =f\left(\lambda \right)d\lambda$$ (7)

**Fig 10:**
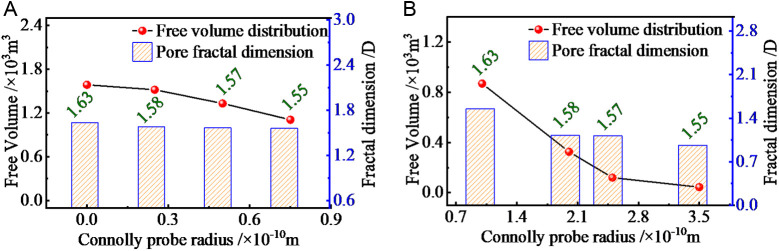
Parameter analysis of the evolution of the molecular structure of bituminous coal within the crystal cell.

Where: $f\left(\lambda \right)=D_{f}\lambda_{\max}^{D_{f}}\lambda^{-\left(D_{f}+1\right)}$ is the probability density of the PSD in fractal porous media, satisfying the normalization condition. The analysis of the evolution parameters of the bituminous coal molecular structure within the cell is presented in Fig 10.

The specific variation of the pore distribution was quantified by calculating the ratio of the spectral peaks of the pores (the ratio of the spectral peak area of the pores to the total spectral peak area) and the ratio of the spectral peaks of the coal matrix (the ratio of the spectral peak area of the coal matrix to the total spectral peak area). The ratio of the spectral peaks of pores and coal matrix was calculated, and it can be seen from the graph that the spectral peak area of coal matrix is much larger than that of pores.

#### (2) Pore permeability characteristics.

Yu *et al.* [39] and Cheng *et al.* [40] obtained an analytical expression for the effective permeability by combining the Hagen-Poiseulle equation and Darcy’s law, assuming that the porous medium consists of a bundle of curved capillaries that follow the fractal scalar law. The fractal permeability *K* can be expressed as,


K=GDfλmax4/GDfλmax4[A(4−Df)]\nulldelimiterspace[A(4−Df)] (8)

Where: *G* = *p*/128 is a geometric constant. Considering the pores in the cross-section as circles of different diameters *k*, the total pore area on the cross-section *A*_p_ can be obtained thus,


$$A=\frac{A_{p}}{\phi }=\frac{1-\phi }{\phi }\cdot \frac{\pi D_{f}\lambda_{\max}^{2}}{4\left(2-D_{f}\right)}$$ (9)

The above equation shows that the permeability is a function of the pore area fractal dimension *D*_f_, and the single cell structure parameters (total cross-sectional area *a*, maximum pore diameter *k*_max_). The permeability considering the fractal dimension of the pore area *D*_f_ is expressed as:


$$K=\frac{2-D_{f}}{32\left(4-D_{f}\right)}\cdot \frac{\lambda_{\max}^{2}\phi }{1-\phi }$$ (10)

Comparisons with other permeability-porosity relationships such as the Kozeny-Carmen equation were conducted (without empirical constants) with a more fundamental physical meaning. The fractal dimension of the pore space, *D*_f_, may be related to the porosity and the maximum pore size, *k*_max_. It is considered that the expressions for permeability can be further extended.

**Fig 11:**
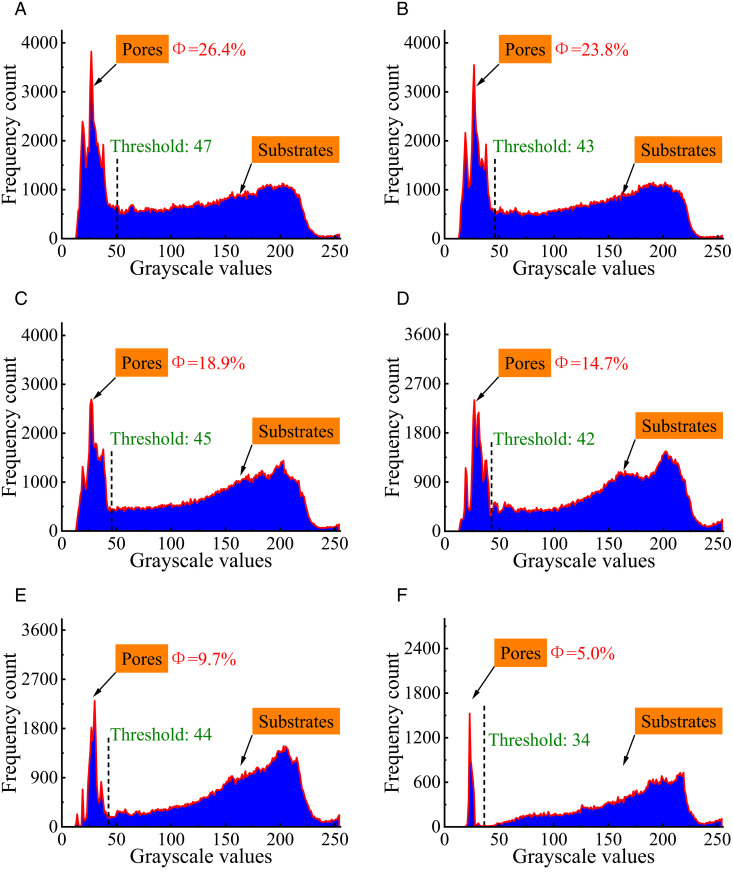
Pore distribution of the molecular structure of the grey-scale treatment. **(a)** Group 1 cut image. **(b)** Group 2 cut image. **(c)** Group 3 cut image **(d)** Group 4 cut image **(e)** Group 5 cut image **(f)** Group 6 cut image.

To quantify the pore distribution of the molecular structure of bituminous coal, the grey-scale analysis of a certain cross-sectional image of this molecule was conducted, and the grey-scale histogram of the analyzed maximum cross-sectional image was derived. The pore distribution of the grey-scale processed molecular structure is shown in Fig 11.

According to the grey histogram, two obvious spectral peaks can be obtained, and the image threshold segmentation point can be judged directly according to whether the probability of the grey value appears abruptly or not, so it is known that the grey value at the valley is the threshold segmentation point. One of the spectral peaks represents the internal pores and the other represents the coal matrix.

As the figure shows, the pore distribution of the molecular structure for the grey-scale treatment covers 26.4%, 23.8%, 18.9%, 14.7%, 9.7%, and 5.0%, respectively. The distribution of the pore structure gradually decreases with the increase of the probe diameter. The threshold splitting points for the pore grey-scale treatment of the molecular structure are 47, 43, 45, 42, 44, and 34, respectively.

### 4.4 Mathematical calculation of pore-permeability

#### (1) Expressions for different porous media materials.

Although the KC equation is widely accepted, it again has many limitations in its use. Therefore, the equation is a semi-empirical relationship. The KC constant is an empirical constant, and previous research indicates that the KC constant is not a constant and may be related to porosity [41]. The KC model is often modified and has different modified versions to improve the estimation of permeability. The KC equations for different porous media are displayed in Table 3.

**Table 3:** KC equation and its modifications for different porous media.

Reference	Permeability equation	Applicable medium
McGregor [42]	$K=\frac{d^{2}}{16c}\frac{\phi^{3}}{{\left(1-\phi \right)}^{2}}$	Textile assembly
Bourbie´ et al. [43]	$K=C\phi^{n}d^{2}$	Porous media
Rodriguez et al. [44]	$K=\frac{\phi^{n+1}}{C{\left(1-\phi^{2}\right)}^{n}}$	Glass and fiber
Mavko and Nur [45]	K=Cd2(ϕ−ϕc)3/Cd2(ϕ−ϕc)3(1+ϕc+ϕ)2\nulldelimiterspace(1+ϕc+ϕ)2	Sanstone carbonate
Bayles et al. [46]	$K=C\frac{\phi^{2+n}}{{\left(1-\phi \right)}^{2}}$	Fine particle filter cakes
Costa [47]	K=Cϕn/Cϕn1−ϕ\nulldelimiterspace1−ϕ	Fiber mats vesicular rocks

Note: *C* is a permeability factor, *n* denotes an empirical exponent, and $\phi_{c}$ is percolation threshold.

#### (2) Expression for calculating the permeability of bituminous coal.

**Fig 12:**
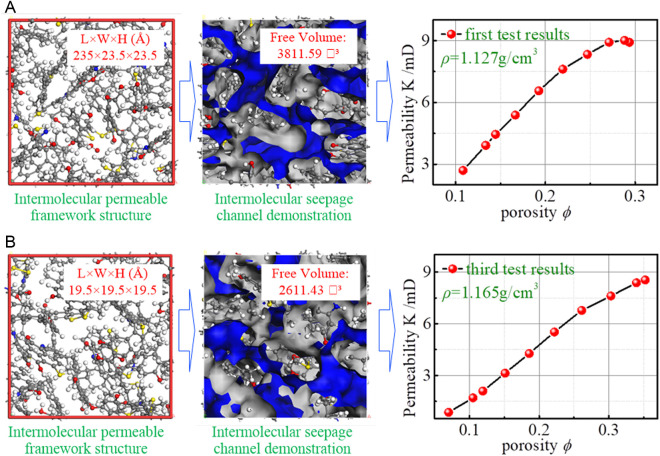
PSD of cells at different densities. **(a)** The result of the first structural optimization **(b)** The result of the second structural optimization **(c)** The result of the third structural optimization.

Numerous theoretical models, experiments, and numerical calculations have shown that the KC equation does not yield a constant value of the KC constant, which instead depends on the microstructure of porosity, pores, and capillaries [48]. The PSD of the cells at different densities is depicted in Fig 12.

The effective stress can better reflect the stress of aggregate particles during the percolation process, and it directly determines the actual stress state of coal particles. All the specimens of each group were analyzed and calculated to ascertain the relationship between porosity and permeability, and the fitted relationship equation is depicted in Table 4.

**Table 4:** Fitting function and correlation.

Sample	Density (g/cm^3^)	Number of test sets	Formula	*R* ^2^
Alpha −1	1.127	First group	*y *= 143 *x*^2.7^/(1 + 13 *x*^2.7^)	0.997
Alpha −2	1.156	Second group	*y *= 114 *x*^1.7^/(1 + 6 *x*^1.7^)	0.994
Alpha −3	1.165	Third group	*y *= 228 *x*^2.1^/(1 + 19 *x*^2.1^)	0.999

Regression analysis was used to characterize the relationship between porosity and permeability as *y* = *ax*^n^/(1 + *cx*^n^), and the goodness of fit is acceptable. By normalization: *K* = 161 ϕ ^2.1^/(1 + 12 ϕ ^2.1^). For this molecular-level porous medium, porosity is the main factor affecting its permeability, and the permeability *K* decreases with the increase of having porosity, indicating that the action of external forces can make some pores and fracture structures inside the coal body to close, and the number of seepage channels is also decreasing, which directly leads to the sharp decrease in the permeability of the fractured coal body.

#### (2) Testing the pore-permeability calculation model.

**Fig 13:**
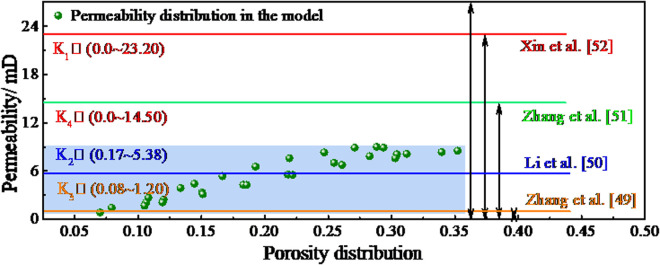
Distribution of bituminous coal permeabilities in the literature.

To test the rationality of the equation, a summary analysis was conducted for the study of porosity and permeability of bituminous coal by reviewing the available literature. The permeability of bituminous coal specimens given in the literature and pertaining to porosities of less than 0.45 was selected, and the reasonableness of the model was then illustrated by comparing the magnitude and distribution of the permeabilities (Fig 13).

The permeability interval of bituminous coal given by Zhang *et al.* [49] is (0.08 ~ 1.20) mD, the permeability interval of bituminous coal given by Li *et al.* [50] is (0.17 ~ 5.38) mD, and the permeability interval of bituminous coal given by Xin *et al.* [51] is (0.0 ~ 23.20) mD. The pore-permeability model established herein has a permeability distribution of (0.87 ~ 9.01) mD, consistent with the distributions mentioned elsewhere. This also shows that the structure of the model calculation is reasonable and can be used to calculate the permeability of bituminous coal.

The pore-permeability calculation model of bituminous coal given in this study uses the calculation principle enshrined in the KC equation to establish the link between porosity and permeability, which can give the permeability of coal directly by molecular modelling. This method simplifies the tedious steps of downhole actual measurement of permeability of such coal and represents a new method for the determination of bituminous coal permeability.

## 5 Conclusion

The permeability determination test of bituminous coal molecules was conducted by independent optimization to investigate the effect of density on the molecular pore surface. The relationship between pore and permeability was revealed and its calculation model was given. Finally, the rationality of the formula was tested. The main conclusions were drawn as follows:

1) Structural models of bituminous coals with three densities, the initial densities of 1.0 g/cm^3^, 1.2 g/cm^3^, and 1.4 g/cm^3^ were used in three sets of tests. The same annealing and molecular dynamics simulations were used for the models to optimize the structures, and the final densities of these three bituminous coals are 1.127 g/cm^3^, 1.156 g/cm^3^, and 1.165 g/cm^3^, respectively. The maximum surface area in the test results is 4.935 × 103 Å^2^, which is the maximum surface area among the three density distributions measured;
2) As the density of bituminous coal molecules increases, the peak value of the PSD curve increases, and the maximum value of 0.29 occurs near the pore size of 2.01 Å. The values of the PSD curves increase with increasing density before the peak and decrease with increasing density beyond the peak. This indicates that the proportion of pores in the oxidized bituminous coal increases at the smaller pore sizes and decreases for larger pores as the density increases.
3) To quantify the pore distribution of the molecular structure of coal, the microstructure of bituminous coal molecules was statistically analyzed using the theory of fractal geometry, and the grey-scale analysis of a certain cross-sectional image of this molecule was conducted; the grey-scale histogram of the cross-sectional image was derived, and the maximum value of the pore distribution of the grey-scale processed molecular structure was 26.4%;
4) For this molecular-level porous medium, porosity is the main factor affecting its permeability, and the permeability decreases with increasing porosity, which can be used to characterize the relationship between porosity and permeability by using: *K* = 161 ϕ ^2.1^/(1 + 12 ϕ ^2.1^).

## Supporting information

S1 File Supporting information. (ZIP)
